# Rapid Alloy Development of Extremely High-Alloyed Metals Using Powder Blends in Laser Powder Bed Fusion

**DOI:** 10.3390/ma12101706

**Published:** 2019-05-26

**Authors:** Simon Ewald, Fabian Kies, Steffen Hermsen, Maximilian Voshage, Christian Haase, Johannes Henrich Schleifenbaum

**Affiliations:** 1 Chair of Digital Additive Production, RWTH Aachen University, 52074 Aachen, Germany; steffen.hermsen@dap.rwth-aachen.de (S.H.); MAXIMILIAN.VOSHAGE@dap.rwth-aachen.de (M.V.); JOHANNES.HENRICH.SCHLEIFENBAUM@dap.rwth-aachen.de (J.H.S.); 2 Steel Institute, RWTH Aachen University, 52072 Aachen, Germany; Fabian.Kies@iehk.rwth-aachen.de (F.K.); christian.haase@iehk.rwth-aachen.de (C.H.); 3 Fraunhofer Institute for Laser Technology, 52074 Aachen, Germany

**Keywords:** additive manufacturing, laser powder bed fusion, high-entropy alloys, multi-principal element alloys, powder blends, rapid alloy development

## Abstract

The design of new alloys by and for metal additive manufacturing (AM) is an emerging field of research. Currently, pre-alloyed powders are used in metal AM, which are expensive and inflexible in terms of varying chemical composition. The present study describes the adaption of rapid alloy development in laser powder bed fusion (LPBF) by using elemental powder blends. This enables an agile and resource-efficient approach to designing and screening new alloys through fast generation of alloys with varying chemical compositions. This method was evaluated on the new and chemically complex materials group of multi-principal element alloys (MPEAs), also known as high-entropy alloys (HEAs). MPEAs constitute ideal candidates for the introduced methodology due to the large space for possible alloys. First, process parameters for LPBF with powder blends containing at least five different elemental powders were developed. Secondly, the influence of processing parameters and the resulting energy density input on the homogeneity of the manufactured parts were investigated. Microstructural characterization was carried out by optical microscopy, electron backscatter diffraction (EBSD), and energy-dispersive X-ray spectroscopy (EDS), while mechanical properties were evaluated using tensile testing. Finally, the applicability of powder blends in LPBF was demonstrated through the manufacture of geometrically complex lattice structures with energy absorption functionality.

## 1. Introduction

Additive manufacturing (AM) is an emerging production technology with enormous potential to replace and supplement conventional manufacturing processes. Especially in the field of metal AM, recent developments in equipment as well as improved part quality have allowed advancements from rapid prototyping of single pieces to final part production. Among the various powder-based metal AM techniques, laser powder bed fusion (LPBF) is currently the most widely used method, as it allows for higher geometrical flexibility than laser metal deposition (LMD) and higher resolution compared to electron beam melting (EBM) [1]. Geometrical freedom, reduced material waste, energy usage, and high degrees of automation are additional advantages of LPBF that contribute to meeting global challenges such as increased individualization, environmental friendliness, and digitalization. Furthermore, elemental segregation can be strongly reduced due to high cooling rates, which makes a large spectrum of materials processable [1,2,3,4,5].

Whereas the various AM techniques enable high degrees of freedom in geometrical design, the methods are rather inflexible with respect to material inputs. So far, mostly pre-alloyed powders or powder blends consisting of two similar materials, e.g., Ti and TiC or TiB, have been utilized to guarantee process stability and chemical and microstructural homogeneity in reproducible properties. Additive manufactured TiC/TiB-reinforced Ti matrix nanocomposites have been used in biomedical applications [6,7,8]. The use of diversified powder blends would open up new degrees of freedom for powder-based AM, especially in alloy design. Utilizing mixtures of multiple powders would allow for fast and simple variations in chemical composition, which would enable the rapid design and screening of new alloys. Therefore, powder-based AM techniques in combination with powder blends might be a new solution for rapidly designing and screening chemically complex materials such as multi-principal element alloys (MPEAs), also known as high-entropy alloys (HEAs) [9,10,11].

MPEAs are a relatively new class of alloys, which instead of relying on one base element contain at least three principal elements with fractions of 5–35 at% each [9,11,12]. Hence, a vast space for chemical compositions and properties is possible, which has stimulated intensive research in this field. With this concept, mechanical and functional property combinations that cannot be found in conventional alloys may be achievable. These property combinations can be varied within a wide range by varying the concentration of one or more of the elements [9,10,11,13]. 

Earlier studies carried out by Haase et al. [14] introduced a robust methodology utilizing thermodynamic modeling and rapid screening using LMD for new MPEAs. The methodology is based on using powder blends as an input material to enable rapid and resource-efficient screening of MPEAs. However, LMD is very limited with respect to producing parts with complex geometry and is prone to defects, which motivates the application of LPBF instead [15]. The possibility of using powder blends as an input material for LPBF has been successful in various previous studies [16,17]. For example, the mechanical properties of high-manganese steels were adjusted by adding elementary Al powder to pre-alloyed steel powder [16,18,19]. However, this approach was not tested with powder blends consisting of more than two powders or different powder morphologies from their production processes (e.g., inert gas atomization, water atomization, grinding).

The goal of the present study is the application of the rapid alloy development methodology introduced by Haase et al. [14] for MPEAs in the LPBF process. First, the Al-C-Co-Fe-Mn-Ni system was exemplarily chosen as the considered material in the present study. This chemically complex system demonstrated an extreme case for powder blends in LPBF, since a variety of different elemental powders, both metallic and nonmetallic, with differing morphologies were used. Secondly, a base composition without C was qualified for the LPBF process to produce fully dense parts. Different energy densities were selected to investigate the homogeneity of the produced samples. Thirdly, the fabricated alloys were evaluated regarding their processability, microstructure, and mechanical properties by using optical microscopy, electron backscatter diffraction (EBSD), energy-dispersive X-ray spectroscopy (EDS), and tensile testing. Finally, parts with complex geometry, i.e. lattice structures, were produced to demonstrate the applicability of powder blends in LPBF. The lattice structures were subsequently compression-tested to evaluate their energy absorption capability. Based on the findings, the correlation between process, microstructure, and mechanical properties is discussed, and the application of elemental powder blends in LPBF is critically evaluated.

## 2. Materials and Methods

### 2.1. Rapid Alloy Development Methodology Using Powder Blends in LPBF

**Figure 1:**
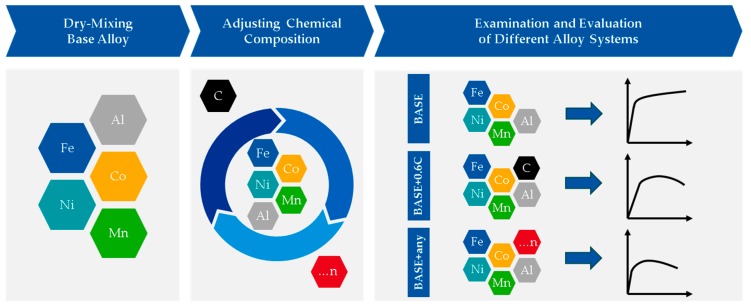
Schematic illustration of the rapid alloy development methodology using powder blends for the laser powder bed fusion (LPBF) process, where “...n” is a placeholder for additional elements.

In Figure 1, the rapid alloy development methodology using powder blends in LPBF of the present study is schematically shown. The approach was divided into three steps. First, a base alloy was created by dry-mixing elemental powders. Secondly, to screen the alloy system, the base alloy was adapted by adding additional elemental powders, e.g., C. Finally, the various created powder blends were processed by LPBF and evaluated by microstructure analysis and mechanical properties.

### 2.2. LPBF Processing

**Figure 2:**
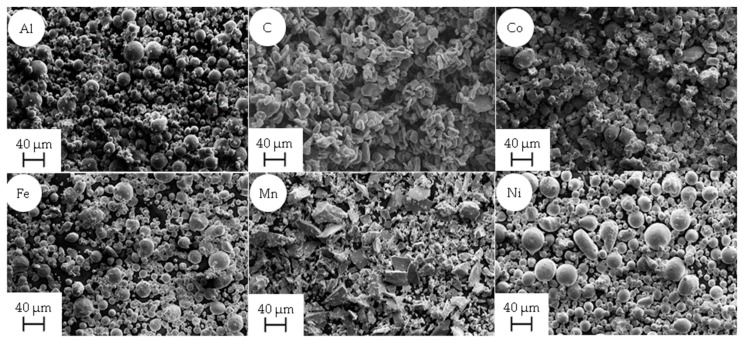
Secondary electron (SE) micrographs showing the different morphologies of the used powders: (**a**) Al, (**b**) C, (**c**) Co, (**d**) Fe, (**e**) Mn, and (**f**) Ni.

The morphology and powder characteristics of the different elemental powders are shown in Figure 2 and Table 1, respectively. The used elemental powders had a purity >99.6 wt% of the respective element. To achieve homogeneously mixed powder blends for the LPBF process, the elemental powders were mixed for 45 min in a Turbula 2F tumbler mixer (Willy A. Bachofen AG, Basel, Switzerland). The two investigated alloy combinations are shown in Table 2. The base alloy was defined as equiatomic CoFeMnNi with the addition of 3 wt% Al (referred to as BASE or Al_0.26_CoFeMnNi in at%) and was further alloyed with 0.6 wt% C (referred to as BASE + 0.6C or C_0.12_Al_0.26_CoFeMnNi in at%). An LPBF process parameter qualification was carried out for BASE and was then transferred to BASE + 0.6C.

The LPBF experiments were performed on an AconityMINI system designed by Aconity3D (Herzogenrath, Germany), which was specifically developed for laboratory use. This system is characterized by a small building space (diameter = 50 mm, with a height of 200 mm) to reduce powder consumption, because the powder blends cannot be separated again after powder-mixing. The beam source was a single-mode fiber laser (wavelength of 1064 nm) with up to 400 W of power output. Samples for microstructure and process parameter development were synthesized on a C45 substrate plate using a bidirectional scanning strategy with 90° rotations between consecutive layers to keep the vector length constant. A vector length of 5 mm resulted from the used samples’ geometry (cuboids with dimensions of 5 × 5 × 10 mm^3^). The energy input during exposure was controlled by the selected process parameters (laser power ($P_{L})$, layer thickness ($D_{S})$, scanning speed $\left(v_{s}\right)$, and hatch distance $\left(\Delta y_{s}\right)$). The volume energy density (*E_V_*) was calculated by Equation (1) [20]:

(1) $$E_{V}=\frac{P_{L}}{D_{S}\cdot v_{s}\cdot \Delta y_{s}}\;.$$

**Figure 3:**
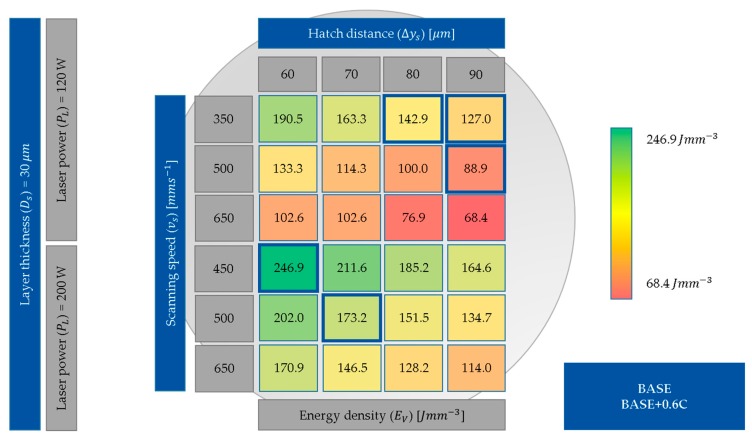
Investigated hatch process parameter combinations with corresponding *E_V_*. The used parameter sets for further samples are marked in blue.

Within the scope of this work, all specimens were manufactured with a constant layer thickness of 30 µm. The investigated process parameter combinations, including the corresponding calculated *E_V_*, are shown in Figure 3. First, the relative densities of the BASE specimens were analyzed. Relative densities above 99.5% were considered to be appropriate for the LPBF process [20]. Based on those results, process parameter sets with low (<100 J mm^−3^), middle (100–200 J mm^−3^), and high (>200 J mm^−3^) *E_V_* were selected to manufacture the BASE and BASE + 0.6C samples.

Samples for mechanical testing were produced on a C45 substrate plate, with a bidirectional scanning strategy, a scanning vector rotation of 33° between consecutive layers, and a scanning vector length of 5 mm. The mechanical properties of the bulk material were investigated with tensile tests. Therefore, cylindrical rods 6 mm in diameter and 60 mm in length were manufactured. For lattice compression tests, f_2_cc lattice structure specimens were manufactured [21] with 10 × 10 × 14 cells, a cell width of 3 mm, dimensions of 30 × 30 × 42 mm^3^, and a strut diameter of 500 µm.

### 2.3. Sample Preparation and Characterization Techniques

Specimens for microstructure analysis were mechanically removed from the baseplate and ground with up to 1200 SiC grit paper followed by polishing using 6 and 1 µm diamond suspension on a plane parallel to the build-up direction. Furthermore, samples were electrolytically polished using a voltage between 25 and 30 V (depending on the chemical composition) for 15 s in A2 electrolyte (Struers, Birmensdorf, Switzerland). Various etchants were tested to make microstructural features visible for optical microscopy. Reasonable results were only obtained with V2A etchant (a mixture consisting of equal parts water and hydrochloric acid with 5% nitric acid) at 70 °C.

Scanning electron microscopy (SEM), energy dispersive X-ray spectroscopy (EDS), and electron backscatter diffraction (EBSD) were performed on a field emission gun (FEG) SEM (Zeiss Sigma, Jena, Germany) with EDS and EBSD detectors (Oxford Instruments, Tubney Woods, Abingdon, UK). Combined EDS and EBSD area mappings were recorded with the voltage at 15 kV with the high-current mode enabled, a working distance between 8.5 and 9 mm, and a step size of 0.2 µm. Analysis and noise reduction of the data were carried out with the MATLAB®-based MTEX toolbox [22,23]. 

The manufactured cylinders were machined to obtain round dog bone tensile specimens with B4 × 20 dimensions after DIN 50125. The mechanical properties were then determined through quasi-static uniaxial tensile tests on a Z4204 device (Zwick/Roell, Ulm, Germany) at room temperature and a strain rate of 2.5 10^−4^·s^−1^. The lattice structures were removed from the baseplate by electrical discharge machining and were then compression-tested at room temperature on a servo-hydraulic universal mechanical testing machine (Schenk, Darmstadt, Germany) equipped with a 400 kN load cell and a constant strain rate of 10^−3^ s^−1^. The specimens were tested according to DIN 50134:2008 and were interpreted after Tancogne et al. and Ashby et al. [24,25]. The specific energy absorption (*E_s_*_40%_) was used to evaluate energy absorption potential, which was calculated by integrating the force–displacement curve up until 40% compression (*E*_40%_) and dividing by the respective weight of the lattice structure [18,26].

## 3. Results

### 3.1. Process Development

**Figure 4:**
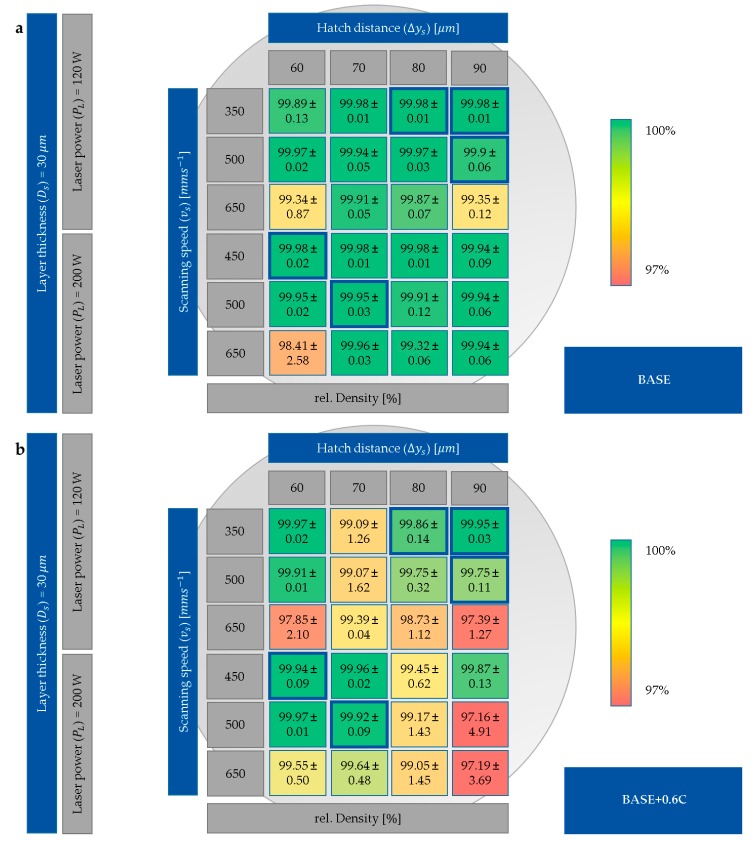
Color-coded relative densities dependent on different process parameters for (**a**) BASE and (**b**) BASE + 0.6C. The selected parameter sets for further production of samples are marked in blue.

The measured relative densities corresponding to the calculated *E_V_* in Figure 3 for BASE and BASE + 0.6C are shown in Figure 4. The densest specimens were manufactured with a laser power *P_L_* of 120 W, a scanning speed *v_s_* of 350 mm·s^−1^, and hatch distances between 70 and 90 µm. The other area of the dense samples was manufactured with a *P_L_* of 200 W, *v_s_* of 450 mm·s^−1^, and hatch distances between 60 and 80 µm. The parameter sets for further sample production were selected accordingly and are marked in Figure 4.

### 3.2. Meltpool Size Depending on the Energy Input

**Figure 5:**
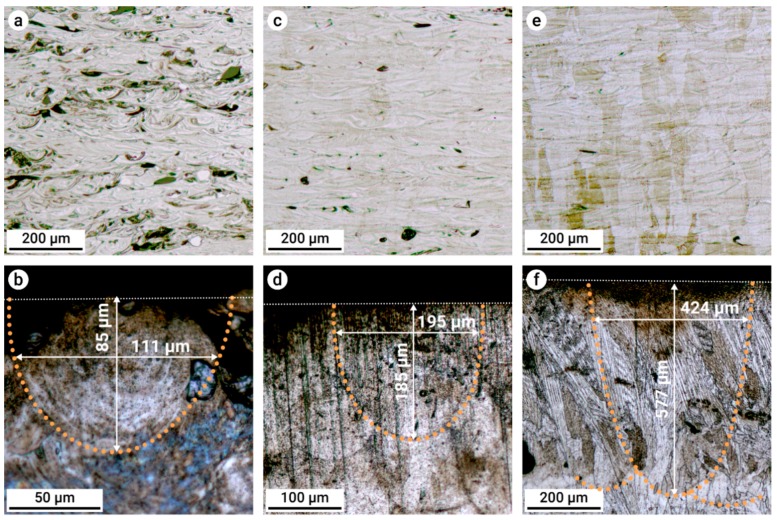
Optical micrographs of etched BASE alloy samples manufactured with energy densities of (**a**,**b**) 68, (**c**,**d**) 173, and (**e**,**f**) 247 J mm^−3^. Microstructure development is shown (a,c,e) in the center of the sample and (b,d,f) on the uppermost layer of the respective samples. White and orange dotted lines denote the sample surface and the shape of the former melt pool, respectively.

An analysis of the microstructure using optical microscopy on selected samples with high relative densities (see Figure 4) is shown in Figure 5. In the micrographs, inhomogeneous regions from partially or unmelted powder particles were more intensely etched, resulting in black regions in the images. With increasing energy densities from 68 to 173 to 247 J mm^−3^ (Figure 5a,c,e), the amount of inhomogeneity was reduced, where the sample was fully homogeneous and uniformly etched with the highest energy density. To show the development of the melt pools in these samples, the uppermost layers of the respective specimens are shown in Figure 5b,d,f. The diameter of the previous melt pool increased from 111 to 195 to 424 µm, while the depth increased from 85 to 185 to 577 µm.

### 3.3. Chemical Homogeneity

**Figure 6:**
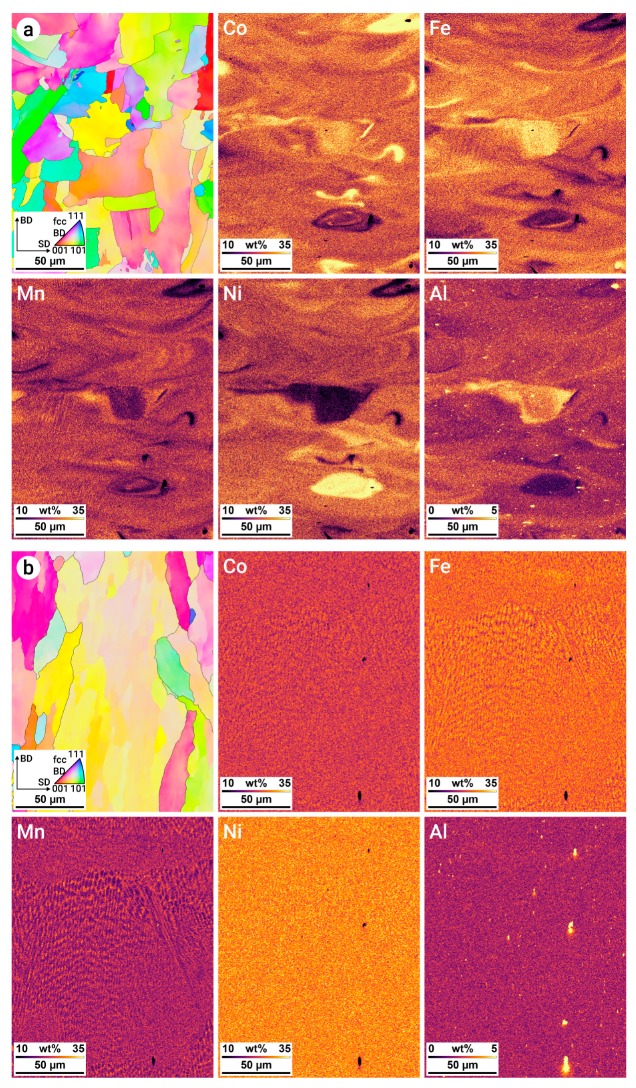
Electron backscatter diffraction (EBSD) and energy-dispersive X-ray spectroscopy (EDS) area maps for the BASE multi-principal element alloys (MPEAs) manufactured with energy densities of (**a**) 143 and (**b**) 247 J mm^−3^. Lower energy densities resulted in regions locally enriched in Co, Fe, and Ni, while higher values produced a more homogeneous elemental distribution. The color-coding of the EBSD maps refers to an inverse pole figure with the build-up direction (BD) as a reference axis.

The chemical homogeneity after processing with different energy densities is shown in Figure 6 with EBSD and EDS area maps. At an energy density of 143 J mm^−3^ (Figure 6a), multiple areas of the microstructure were strongly enriched in Co, Fe, and Ni below the prior melt pool boundaries. These were caused by partially melted powder particles during LPBF processing. When the energy density was sufficiently high, a more homogeneous elemental distribution was obtained (Figure 6b), where slight local differences in composition resulted from dendritic solidification. A low fraction of Al-oxides can be observed in Figure 6, where the dots represent oxide clusters.

### 3.4. Mechanical Properties

**Figure 7:**
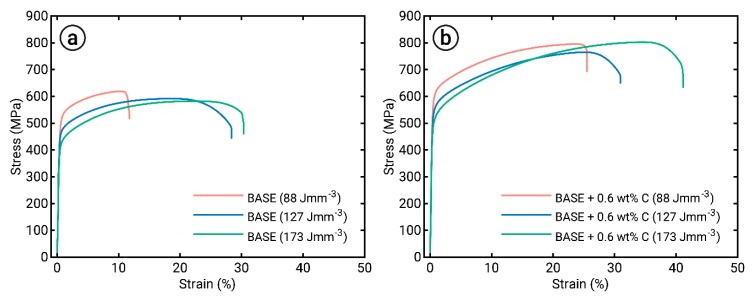
Engineering stress–strain curves of the (**a**) BASE and (**b**) BASE + 0.6C alloys processed with the shown energy densities. Total elongation was drastically reduced, whereas strength increased slightly at low energy densities. The addition of 0.6 wt% C led to strongly increased strength and ductility.

The results of tensile tests on samples manufactured with different energy densities are shown in Figure 7. At lower energy densities of 88 and 127 J mm^−3^, both investigated compositions showed drastically reduced elongation and slightly increased strength compared to a higher energy density state of 173 J mm^−3^. Additionally, overall strength and ductility was increased with the addition of 0.6 wt% C to the alloy.

**Figure 8:**
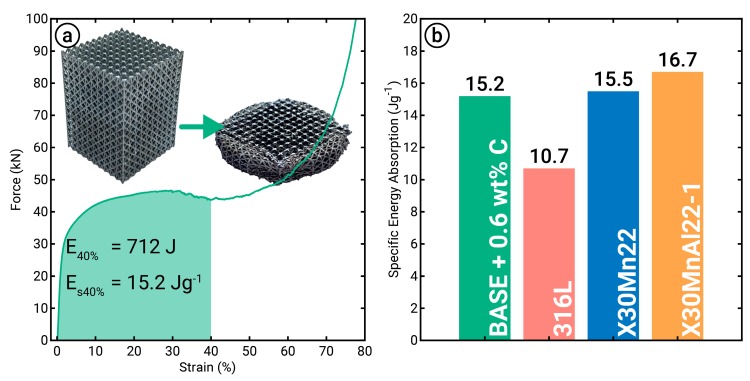
(**a**) Force-engineering strain curve of the BASE + 0.6C lattice structure during compression testing. The sample was manufactured using an energy density of 173 J mm^−3^. (**b**) Comparison of the specific absorbed energy to lattice structures [18] of the same geometry (f_2_cc_z_ type, 500 µm struts, 3 mm cell width) but different material.

Based on the mechanical properties from tensile testing (Figure 7), the alloy and processing parameters with the highest combinations of strength and ductility were used to manufacture the lattice structure. Consequently, BASE + 0.6C was chosen at an energy density of 173 J mm^−3^. The corresponding force–strain curve obtained by compression testing of the produced lattice structure and comparisons with other materials are shown in Figure 8. After yielding, the force increased to 46 kN at around 30% compression. Further loading led to decreased force, indicating the failure of some load-bearing struts in the structure. At around 60% compression, the compaction region was reached, where the lattice was fully compressed. With 40% strain, the lattice structure absorbed an energy of 712 J (*E*_40%_), resulting in a specific energy absorption of 15.2 J g^−1^ when taking the weight of the specimen into account. Compared to lattice structures with the same geometry but a different alloy, the energy absorption of the MPEAs was higher than that of 316L, while it was on a similar level to high-Manganese steels.

## 4. Discussion

In the following, the adaption of the rapid alloy development methodology from LMD to LPBF as well as the processability of powder blends and the influence of process parameters on the mechanical properties are discussed.

After process parameter optimization, several parameter sets with relative densities above 99.5% and a homogenous elemental distribution were determined for BASE and BASE + 0.6C (Figure 4, Figure 5 and Figure 6). These results showed the successful transition of the rapid alloy development methodology first applied in LMD [14] to LPBF. With LPBF, less elemental segregation takes place during solidification due to the higher cooling rates compared to LMD [18,27]. Therefore, the possibility of processable materials is larger than in LMD, and better mechanical properties can be achieved because of grain refinement [28,29,30]. However, the powder consumption in LPBF is higher compared to LMD due to the necessity of filling the building chamber. With respect to flexible alloy modification, LMD enables in-situ mixing of various powders, chemical gradients within specimens/parts, and efficient powder consumption [1,31]. Overall, LPBF is more suitable for alloy design due to higher reproducibility and lower defect density compared to LMD.

The powder blends utilized in the process contained up to six different elemental powders with varying morphology. The Ni, Al, and Fe powders were inert gas-atomized, which is the most used production technology for the synthesis of powders used in LPBF. The characteristics of inert gas-atomized powders are a spherical shape, high flowability, and high purity [32]. Mn and C were produced by grinding, resulting in flake-shaped particles with poor flowability and thus poor recoating behavior during LPBF [32]. The Co powder was water-atomized and revealed an irregular splash-shaped morphology. Nevertheless, the Co powder still retained reasonable flowability. The bulk density of the powders was in the range between 0.544 g ml^−1^ (C) and 4.545 g ml^−1^ (Ni). The particle size distribution (PSD) for all powders was in the range of 10 and 45 µm, except for Mn and C, where particles with a diameter below 10 µm were present, which reduced the flowability and thus influenced powder recoating behavior. The flowability (defined by the average avalanche angle) was between 48° for Co and 62° for C. As evidenced by the high relative densities achieved (Figure 4), the powder blends revealed a suitable recoating behavior. Therefore, the poor flowability of powders (C, Mn) could be compensated for by blending them with powders characterized by high flowability (Fe, Ni, and Al).

The absorption tested for the used wavelength of 1064 nm was determined to be 48% for Al and up to 90% for C. Therefore, the energy input into the powder varied significantly between the different powders. This could cause overheating of powders with higher absorption as well as incomplete melting of powders with low absorption. However, once the melt pool was fully developed, the absorption remained constant and the influence of the different absorption coefficients vanished due to a preflowing melt pool [33]. The use of elemental powders led to higher evaporation, since the elements with lower melting temperatures melted first and could overheat, whereas the elements with higher melting temperatures showed delayed transformation into the liquid phase [34] (Table 3). Consequently, Mn and Co had the highest and lowest evaporation rates due to their evaporation temperatures, respectively. This behavior was validated by EDS mapping, as shown in Figure 6. The used elemental manganese powder showed high oxidation and therefore a high oxygen content due to the production process. The oxygen introduced by the manganese powder resulted in the formation of Al-oxides due to the high affinity of Al to oxygen [35]. A low fraction of Al-oxides could be observed in the EDS element mapping, as shown in Figure 6. The Al-oxide clusters are shown as small white dots in the aluminum mapping. Nevertheless, an overall homogenous element distribution could be observed in the LPBF-produced samples using powder blends. This proved in general the applicability of powder blends in LPBF, even for complex powder blends consisting of up to six different powders with varying morphologies and PSDs.

The results showed that dense parts could be produced using a broad spectrum of process parameters. This allowed for the application of a wide processing window, with *E_V_* in the range between 60 and 240 J mm^−3^ for the investigated alloying system. A higher energy input resulted in higher homogeneity of the elemental distribution due to enlargement of the melt pool size by means of width and depth, which resulted in a higher number of powder particles in the melt pool [36]. The calculated particle amount contained in the melt pools shown in Figure 5 was 15 for Figure 5b, 57 for Figure 5d, and 386 for Figure 5f. This enlarged melt pool enabled the enhanced mixing of elements and at the same time facilitated the remelting of previously solidified layers. Hence, the higher the energy density was, the higher the homogeneity of the elemental distribution due to complete melting and mixing of the various elemental powder particles was. In Figure 6a, partially molten particles and heterogeneously composed areas can be found. Furthermore, the increased energy input also promoted higher homogeneity due to a higher melt pool temperature [36]. As a consequence, less nonfused material was observed (Figure 5 and Figure 6). 

Homogeneity also affected the mechanical properties. Whereas specimens produced with a higher *E_V_* resulted in increased elongation, samples manufactured with a lower *E_V_* revealed reduced fracture strain (Figure 7). Presumably, the loss of ductility was associated with locally heterogeneous deformation behavior, as well as notch effects in the vicinity of unmolten particles [37]. Since the process development allowed for the production of samples with similarly high relative densities, the detrimental effects of the remaining porosity could be ruled out as a cause of reduced ductility. However, it must also be noted that very high energy densities may result in pronounced evaporation/gas entrapment and thus more gas pores [38]. These pores usually contribute to deteriorated mechanical properties [39]. Hence, an optimum process window avoids the effects associated with very low and very high input energies. 

Furthermore, the rapid alloy development methodology introduced was used for material screening. As an example, C was added to the BASE alloy to tailor the mechanical properties. C was chosen because it is known as a very efficient solid solution strengthening element in fcc alloys. By adding 0.6 wt% C, the mechanical properties of the BASE alloy were improved with respect to both strength and ductility (Figure 7). To exclude further microstructural modifications, the process parameters were kept constant for both BASE and BASE + 0.6C. A usable parameter set was determined within the process parameter development (Figure 3 and Figure 4). The development showed similar results for relative density for both alloys using the same process parameter sets. Nevertheless, a negligible influence of the process parameters on the relative density was determined. Surprisingly though, the application of powder blends containing elemental C powder with poor flowability and flake-shaped morphology did not deteriorate processability. Furthermore, pure C particles and large carbides were not observed, which suggested the complete dissolution and homogeneous distribution of interstitial C atoms. Therefore, the usage of complex powder blends to tailor material properties to design materials by and for AM can be confirmed. 

To demonstrate the potential to produce geometrically complex structures with powder blends, f_2_cc_z_ lattice structures were synthesized. These delicate structures are a suitable measure for the reliability of the material produced from powder blends due to the sensitivity of the thin struts to material homogeneity. For instance, most common strut sizes for LPBF-manufactured lattice structures are 500 µm in diameter [21,40], which means that a laser beam melts approximately 350 particles on the top layer per strut. An energy input of 173 J mm^−3^, considered to be the optimal value for the BASE + 0.6C alloy, was chosen, resulting in ductile and homogeneous deformation behavior (Figure 8). Compared to the results of Kies et al. [18], where high-manganese transformation-induced plasticity (TRIP) and twinning-induced plasticity (TWIP) steels were tested, a similar specific energy absorption was achieved. Furthermore, compared to the benchmark material 316L, the specific energy absorption of the tested MPEAs was increased by approximately 75% [18,31]. Therefore, the usability of the rapid alloying methodology was additionally validated due to successfully manufacturing lattice structure specimens with reliable deformation behavior.

## 5. Conclusions

The present study showed that fully dense and chemically homogenous MPEAs could be successfully manufactured by using elemental powder blends in LPBF. This enables a rapid and resource-efficient approach to screen and design novel materials. The following conclusions could be drawn:

1. Complex powder blends consisting of up to six elemental powders with different morphologies, size distributions, and amounts could be applied to the LPBF process. Therefore, rapid alloy development of chemically complex metallic alloys is possible, which was demonstrated on C-Al-Co-Fe-Mn-Ni MPEAs. Compared to other metal AM processes, higher cooling rates facilitated improved material properties, e.g., high strength, high energy absorption capacity, and less elemental segregation.
2. Chemical homogeneity was strongly dependent on the energy input and resulting size of the melt pool formed during LPBF. On the one hand, insufficient energy input resulted in inhomogeneous elemental distribution, as powders with high melting points were only partially melted. On the other hand, small melt pool sizes prohibited sufficient elemental mixing. Optimal energy input resulted in alloys with high chemical homogeneity.
3. The mechanical properties of the investigated Al_0.26_CoFeMnNi system were significantly improved by the addition of 0.6 wt% C, resulting in both increased strength and ductility. Therefore, the methodology of combining powder blending and LPBF was proven to be a promising method to produce high-quality material containing significant nonmetallic additions, such as C.
4. LPBF using powder blends enables manufacturing of parts with complex geometry, e.g., lattice structures, and reliable mechanical properties. The produced lattice structures indicated a higher energy absorption capacity compared to the commonly used 316L and were comparable to high-Manganese steel samples.

## Figures and Tables

**Table 1:** Overview of the characteristics of the different elemental powders.

Powder	Manufacturing Method	Particle Size Distribution (μm)	Form	Flowability as Avalanche Angle (°)	Laser Absorption for 1064 nm (%)	Bulk Density (g·ml^−1^)
Al	gas-atomized (Ar)	10–45	spherical	58 ± 0.18	48 ± 0.33	1.31 ± 0.02
C	ground	up to 45	flake-shaped	62 ± 0.18	90 ± 0.29	0.54 ± 0.02
Co	water-atomized	15–45	splash-shaped	48 ± 0.10	73 ± 0.06	3.40 ± 0.02
Fe	gas-atomized (Ar)	10–45	spherical	56 ± 0.18	74 ± 0.65	4.03 ± 0.03
Mn	ground	up to 45	flake-shaped	52 ± 0.09	73 ± 0.09	2.50 ± 0.02
Ni	gas-atomized (Ar)	15–45	spherical	55 ± 0.19	65 ± 0.34	4.55 ± 0.02

**Table 2:** Nominal chemical composition of the investigated alloys.

Alloy	Element	Al	C	Co	Fe	Mn	Ni
BASE	(at%)	6.14	–	23.46	23.46	23.46	23.46
	(wt%)	3.00	–	25.03	23.72	23.33	24.93
BASE + 0.6C	(at%)	6.01	2.70	22.82	22.82	22.82	22.82
	(wt%)	3.00	0.60	24.87	23.57	23.19	24.77

**Table 3:** Evaporation and melting temperatures of the used elements [19,20].

Element	Al	C	Co	Fe	Mn	Ni
Melting temperature (°C)	660	–	1495	1538	1246	1455
Evaporation temperature (°C)	2470	4827	2870	2862	2061	2732
